# Essential Oil-Containing Mouthrinses for Plaque and Gingivitis Control: Formulation Chemistry, Human Salivary Biomarkers, Mechanistic Evidence, and Research Priorities

**DOI:** 10.3390/molecules31193417

**Published:** 2026-09-25

**Authors:** Hubert Sytykiewicz

**Affiliations:** Institute of Biological Sciences, Faculty of Natural Sciences, University of Siedlce, 14 Prusa St., 08-110 Siedlce, Poland; hubert.sytykiewicz@uws.edu.pl

**Keywords:** essential oil-containing mouthrinses, human saliva, salivary biomarkers, plaque, gingivitis, periodontal health, formulation chemistry

## Abstract

Selected essential oil (EO)-containing mouthrinses improve plaque and gingivitis control when used alongside mechanical oral hygiene, but their molecular effects in human saliva remain poorly defined. This narrative review integrates formulation chemistry, oral exposure, human salivary evidence, and mechanistic studies. Three studies identified in this review evaluated salivary immunoglobulin A, immunoglobulin G, or interleukin-1β. Differences in populations, co-interventions, sampling, and analytical methods limit comparison and do not establish reproducible modulation of a single biomarker. Other human studies assessed salivary flow, pH, proteomic responses, microbial counts, nitrate reduction, and oral biofilms. Mechanistic evidence largely derives from isolated constituents in cellular, animal, or computational models. A study of a complete zinc-containing formulation in human oral cells provides formulation-level evidence, but neither this study nor the constituent literature establishes target engagement after mouthrinsing in humans. Future trials should connect chemically characterized formulations and measured oral exposure with target-specific assays, validated salivary endpoints, microbial function, clinical outcomes, and safety. Current evidence supports selected formulations for plaque and gingivitis control; it does not establish an EO class effect or a validated salivary mechanism of clinical benefit.

## 1. Introduction

Whole saliva contains glandular secretions with gingival crevicular fluid (GCF), epithelial material, microorganisms, food-derived compounds, and exogenous exposures. This composite origin permits repeated assessment of salivary physiology, biofilm metabolism, gingival inflammation, bleeding, and tissue breakdown, but it also complicates interpretation [1,2,3,4,5,6,7,8]. A lower analyte concentration may reflect reduced production, greater clearance, increased flow, or analytical interference rather than biological improvement. Salivary measurements after an EO mouthrinse therefore require standardized collection, flow assessment, formulation-specific interference testing, and clinical context. IL-1β and MMP-8 and other inflammatory mediators are widely investigated periodontal biomarker candidates, but none is specific to EO exposure [9,10,11,12,13,14,15,16,17,18]. Attribution requires an incremental effect beyond mechanical therapy, an appropriate comparator, and a plausible temporal relation between exposure, biomarker change, and clinical response.

EO-containing mouthrinses occupy an unusual evidential position. Meta-analyses and long-term trials support selected fixed-dose commercial formulations for plaque and gingivitis control [19,20,21,22,23,24], whereas direct human salivary host-biomarker evidence is sparse. Accordingly, plaque accumulation and gingival inflammation, not oral disease in general, define the clinical scope reflected in the title. The clinical literature evaluates complete formulations, but mechanistic studies often test isolated compounds or complete plant oils at concentrations that have not been demonstrated after rinsing. This review therefore separates three intervention levels (i.e., isolated constituent, complete EO, and formulated mouthrinse) and follows a defined evidence sequence: formulation identity and concentration → local oral exposure → target or functional engagement → biological response → salivary readout → clinical outcome and safety. The objectives were to identify salivary endpoints suitable for EO trials, distinguish direct human biomarker evidence from adjacent and clinical-only evidence, examine oral and non-oral mechanisms at the level demonstrated, and define the measurements needed for causal attribution.

## 2. Review Scope, Literature Identification, and Evidential Rules

The literature search was last updated on 9 September 2026 using PubMed/MEDLINE and the Web of Science Core Collection. Six complementary blocks covered clinical plaque/gingivitis, saliva/biomarkers/physiology, oral microbial and mechanistic evidence, direct molecular targets, host signaling, and a dedicated methyl-salicylate supplementary targeted search. All six Web of Science Core Collection searches were run in the Topic field (TS). The clinical and salivary blocks identified the core human evidence; broader blocks mapped mechanistic context. Exact strings, field syntax, and raw block-level retrieval counts are reported in Supplementary Table S1. Publisher sites, DOI/Crossref metadata, and reference lists were used for full-text retrieval, bibliographic verification, and backward citation tracking, not as independent databases. No language filter or “Humans” filter was applied; study type and experimental model were determined during eligibility assessment. Records published or available online by the cutoff were considered for inclusion.

Eligible human studies evaluated an EO-containing oral formulation or named EO constituent and reported a salivary host marker, salivary physiology or biochemistry, an oral microbial or biofilm endpoint, a GCF marker, a plaque, gingival, or periodontal outcome, or safety outcome. Mechanistic primary studies were included when a named EO, formulation, or constituent was tested in an oral microorganism, a human oral cell or tissue model, another defined biological model, a purified target, or a computational system relevant to a stated oral hypothesis. Systematic reviews and methodological papers were retained for context or assay interpretation. Exclusions included chemically undefined non-EO herbal mixtures, non-oral uses without a direct mechanistic link, duplicate reports, conference abstracts with insufficient methodological detail, and docking-only claims presented as binding or target engagement.

Titles and abstracts were screened iteratively, and potentially eligible reports were assessed in full text. Overlap across blocks and databases was consolidated using PMID, DOI, normalized title, and manual verification; backward citation tracking supplemented retrieval. Because the review linked clinical, salivary, chemical, microbial, and mechanistic domains rather than estimating a pooled effect, broad mechanistic blocks were used for evidence mapping, and their raw counts were not summed as a PRISMA-style screening denominator. The review did not include independent duplicate screening, a PRISMA flow diagram, a formal risk-of-bias tool, certainty grading, or quantitative meta-analysis. Records were assigned to four evidence categories: (1) direct human salivary inflammatory or immune host-biomarker evidence, defined as human intervention studies in which a targeted salivary inflammatory or immune analyte was evaluated as a treatment-response endpoint; (2) adjacent human oral or salivary functional and biochemical evidence, including salivary secretion, pH, general biochemical measures, exploratory proteomic responses, oral microbial or biofilm endpoints, and GCF markers; (3) clinical outcomes without a Category 1 or 2 endpoint; and (4) mechanistic or exploratory evidence. Within Category 4, isolated constituents, complete EOs, and commercial formulations were kept separate, and human oral cells or tissues were distinguished from heterologous, non-oral, animal, microbial, purified-target, and computational models. SANRA was used only as an appraisal checklist for the quality of the completed narrative review; it did not define eligibility and is not presented as a reporting guideline or substitute for the search and selection methods [25].

## 3. Saliva as a Pharmacodynamic Matrix

### 3.1. Biological Origin and Choice of Endpoint

Whole-salivary concentration reflects the combined effects of release, transport, dilution, degradation, and clearance. Periodontal mediators enter through gingival crevicular fluid, inflammatory exudate, bleeding, epithelial shedding, and microbial release, whereas gland-derived proteins and electrolytes vary with stimulation and autonomic tone [3,4,5,6,7]. An EO formulation may change several of these determinants at once. Changes in salivary flow caused by stimulation can complicate interpretation of concentration-based host biomarkers [3,4,5,6,7]; antimicrobial effects may likewise alter biofilm-derived stimulation. Concentration per milliliter alone cannot distinguish these processes. A pragmatic core panel for future periodontal EO trials would be deliberately small. IL-1β provides a clinically interpretable inflammatory readout; active MMP-8 may more closely reflect collagenolytic activity than total immunoreactive MMP-8; and bleeding on probing or a standardized gingival bleeding index supplies the tissue context [9,10,11,12,13,14].

IL-6, TNF-α, and IL-8/CXCL8 are plausible secondary endpoints but may be low, variably detectable, or derived from several oral sources [9,10,11,12]. MMP-9 may provide additional information on matrix remodeling but should be treated as a secondary or exploratory endpoint relative to the more extensively studied salivary MMP-8 [9,10,11,12,13]. Expanding a panel without a prespecified causal model increases multiplicity without necessarily increasing insight. Redox endpoints require paired interpretation. Malondialdehyde (MDA) and 8-hydroxy-2′-deoxyguanosine reflect lipid and DNA oxidation, respectively, but their salivary interpretation is sensitive to systemic, behavioral, and analytical confounding [26,27,28,29,30,31].

Total antioxidant capacity (TAC) is a global functional measure strongly influenced by urate and assay chemistry; superoxide dismutase, glutathione peroxidase, and catalase may rise as compensation or fall with depletion [26,27,28,29,30,31,32,33,34]. A defensible redox panel therefore includes at least one damage marker and one defense marker, together with clinical and microbial measures. DPPH (2,2-diphenyl-1-picrylhydrazyl) or ABTS (2,2′-azino-bis(3-ethylbenzothiazoline-6-sulfonic acid)) radical scavenging activity of an oil is not a substitute for this human evidence. Salivaomics may broaden the analysis beyond a few candidate analytes. Proteomics, small extracellular vesicle analyses, and microbiome profiling may reveal coordinated responses, but discovery is easily confounded by collection, vesicle isolation, protein depletion, batch effects, and unstable normalization [35,36,37,38]. Multi-omics becomes most useful when it tests a temporal model: an early change in microbial function, followed by altered inflammatory mediators and then a clinical response. A single high-dimensional sample collected at the final visit cannot establish that sequence.

Salivaomics may also provide information beyond periodontal monitoring. Chronic periodontal inflammation, microbial dysbiosis, oxidative stress, and genetic or epigenetic dysregulation overlap with processes discussed in relation to early oral cancerization [39]. Salivary inflammatory mediators, microbial signatures, proteins, DNA methylation patterns, and microRNAs are being investigated for risk stratification in this setting [39]. These signals are not established EO-responsive biomarkers and are not specific to malignant transformation; periodontal status and relevant clinical covariates must therefore be considered in interpretation. In EO trials they should be treated as exploratory disease-context or safety measures, not as evidence that a mouthrinse modifies oral-cancer risk.

### 3.2. Pre-Analytical Control and Analytical Interference

Unstimulated and stimulated saliva are not interchangeable. Stimulation changes glandular contribution, pH, bicarbonate, protein concentration, and dilution. Collection time, fasting interval, recent oral hygiene, smoking, exercise, acute stress, collection device, centrifugation, storage temperature, and freeze–thaw history should be prespecified [5,6,7,8,40,41,42,43]. Flow should be reported and, where biologically appropriate, results should be expressed both as concentration and analyte output rate. Clinical periodontal status and visible blood contamination must be documented at each collection. Immediate post-rinse sampling creates a special analytical problem. Residual ethanol, surfactants, flavoring agents, hydrophobic constituents, and altered ionic strength may affect antibody binding, optical readouts, enzyme activity, protein solubility, or mass-spectrometric ionization. Apparent suppression of a biomarker may therefore be analytical rather than biological. Before clinical testing, the actual formulation should undergo spike–recovery, dilution-linearity, hemolysis, and interference testing in pooled saliva. Separate samples are often needed for pharmacokinetic exposure and for delayed pharmacodynamic biomarkers. Assay performance in saliva may be analyte-specific even when the same immunoassay platform is used, so validation should be demonstrated for each endpoint rather than assumed from a kit validated for blood [44]. Normalization should match the question. Total protein normalization may introduce bias when flow, inflammation, or protein secretion is itself changed by chemosensory stimulation. For extracellular vesicles, particle count, vesicle-associated protein, and input volume answer different questions and may produce different conclusions. The normalization strategy should therefore be prespecified and, where feasible, supported by sensitivity analyses [36].

### 3.3. Temporal Sampling and Interpretation of Change

A salivary endpoint can be interpreted as a pharmacodynamic readout only when the sampling interval is biologically plausible (Table 1). Exposure chemistry, acute secretory responses, microbial responses, and downstream host biomarkers should be sampled on prespecified time scales appropriate to the process being measured rather than collapsed into a single post-treatment time point. Combining mechanistically distinct sampling windows obscures temporal interpretation. For mechanistic attribution, a minimum sampling design would include a pre-dose trough sample, an immediate exposure sample reserved for analytical chemistry, and a delayed biomarker sample collected after a validated washout. The direction of change also requires a defined denominator. Changes in salivary analyte concentration should be interpreted together with salivary flow, because variation in flow can alter concentration without a proportional change in analyte output. Where biologically appropriate, concentration and flow should therefore be measured concurrently, and analyte output rate should be reported alongside concentration. Output rate does not fully resolve source attribution, because locally derived gingival mediators do not necessarily scale with glandular flow. Repeated measures within participants are preferable to a single baseline-to-endpoint comparison because they allow assessment of day-to-day reliability and dose timing. Concurrent clinical measurements are indispensable. Whole-salivary IL-1β or active MMP-8 should be interpreted alongside full-mouth bleeding and plaque scores and standardized periodontal case definitions; redox markers require smoking, diabetes, diet, and supplement data. When the hypothesized source is periodontal, paired gingival crevicular fluid (GCF) may test whether the whole-saliva signal tracks local sites. When the hypothesized source is glandular or neural, gland-specific collection or salivary-protein signatures may be more informative. The biological compartment should determine the sampling design, not the convenience of whole saliva alone.

## 4. Formulation Chemistry, Local Exposure, and Translational Linkage

### 4.1. Chemical Identity, Chemotypes, and Analytical Specification

Botanical name alone does not define an EO intervention. Species, chemotype, plant part, geography, extraction, storage, and batch alter the abundance and oxidation state of volatile constituents [47,48,49,50,51,52,53]. Complete EOs, isolated constituents, and commercial mouthrinses should therefore be treated as distinct experimental entities. The fixed-dose formulation used in much of the clinical literature contains eucalyptol (0.092%), thymol (0.064%), methyl salicylate (0.060%), and menthol (0.042%) in alcohol-containing or alcohol-free vehicles [54]. Product-level efficacy cannot be assigned to one constituent without component-controlled comparisons.

Among these constituents, methyl salicylate warrants separate consideration because it is one of the four declared active ingredients in the fixed-dose EO mouthrinse evaluated in much of the clinical literature [54]. However, it has been administered clinically as part of the complete formulation, and the available trials do not establish its independent contribution to plaque or gingivitis control. Component-level in vitro experiments evaluated individual Listerine ingredients and their combinations [55]. These findings do not establish the independent clinical contribution of methyl salicylate to the efficacy of the complete mouthrinse. In a recent oral-cell study, thymol, eucalyptol, menthol, and methyl salicylate tested individually under the authors’ experimental conditions did not reproduce the host-transcriptional effect of a complete zinc-containing Listerine formulation [56]. Those constituent experiments did not constitute an exposure-matched deconstruction of the 5% formulation and therefore do not quantify the contribution of each active constituent. Oral-site exposure and molecular target engagement after rinsing were not identified at the individual-compound level for methyl salicylate. Future studies should quantify methyl salicylate in saliva, plaque, and mucosal or buccal samples and use component-controlled, exposure-matched designs to determine its contribution to the complete formulation.

Plant-specific formulations require batch-resolved gas chromatography–mass spectrometry (GC–MS) or equivalent analysis because EO composition may vary with species, chemotype, plant part, processing, storage, and oxidation state [47,48,49,50,51,52,53]. In the *Lippia sidoides* studies summarized in Table 2, EO concentration and thymol/carvacrol dominance were defining characteristics of the formulation, whereas data on carvacrol, β-caryophyllene, or linalool from isolated-compound or non-oral experiments represent separate evidence levels. For every formulation, the nominal concentration should be distinguished from dissolved, micelle-bound, protein-bound, and free fractions. A vehicle-matched comparator is preferable to water when ethanol, surfactants, flavoring agents, or other excipients may influence sensation, permeability, cytocompatibility, or assay performance.

### 4.2. Structural Determinants, Mixture Effects, and Multitarget Pharmacology

Structural similarity does not establish equivalent oral activity. Thymol and carvacrol share a phenolic monoterpene scaffold but differ in hydroxyl position and mixture behavior [57,58,59,60]. Their clinically tested formulation contexts are summarized separately in Table 2. Eugenol, 1,8-cineole, menthol, methyl salicylate, β-caryophyllene, and linalool likewise differ in polarity, volatility, reactivity, and protein or membrane partitioning. Mechanistic comparison is informative only when concentration, vehicle, cytocompatibility, and target dependence are measured in an exposure-relevant model. Table 2 therefore links each constituent to the clinically tested formulation context and states where evidence is limited to an isolated compound or a non-oral system.

### 4.3. Formulation and Colloidal Delivery

Vehicle and colloidal delivery can alter dispersion, release, oxidation, mucosal contact, and free concentration [61,62,63]. Studies involving nanoemulsions, liposomes, food matrices, skin models, or transdermal delivery may identify formulation principles [61,62,63], but they do not demonstrate delivery through human oral mucosa. Oral studies should report active concentrations, vehicle composition, droplet or vesicle properties where relevant, stability after dilution in saliva, constituent recovery, release, retention, cytocompatibility, and sensory tolerability. These are properties of the complete formulation, not of the plant name alone (Table 2; Figure 1).

**Table 2:** Clinically tested formulation context, nominal concentrations, and mechanistic evidence for representative EO constituents.

Constituent	Clinically Tested Formulation Context/Nominal Concentration	Chemical and Exposure Determinants	Mechanistic Evidence and Experimental Level	Translational Boundary/References
Thymol	Fixed-dose hydroalcoholic and alcohol-free EO rinses: 0.064%; also abundant in *Lippia sidoides* oils.	Phenolic monoterpene; positional isomer of carvacrol; hydrophobic and oxidation-sensitive.	Membrane activity; TRPV3 activation; limited TRPM8 activity; NF-κB/MAPK-associated findings, mainly heterologous or non-oral.	Clinical efficacy pertains to the complete formulation as tested; oral target engagement after rinsing was not identified in this review [19,20,21,22,23,24,59,60,64,65,66,67,68,69,70].
1,8-Cineole (eucalyptol)	Fixed-dose hydroalcoholic and alcohol-free EO rinses: 0.092%.	Volatile cyclic ether; free exposure depends on vehicle, retention, and sampling time.	TRPM8 agonism and TRPA1 antagonism in heterologous systems; NF-κB/JNK nodes in non-oral human cells.	No exposure-matched target engagement in gingival or salivary tissue was identified in this review [67,71,72,73].
Menthol	Fixed-dose hydroalcoholic and alcohol-free EO rinses: 0.042%; isolated menthol used in a human salivation study.	Monoterpene alcohol; stereochemistry, volatility, and flow-mediated dilution affect response.	Direct TRPM8 pharmacology; human oral exposure increased salivary flow and altered proteomic output.	Supports chemosensory physiology but not an anti-inflammatory mouthrinse mechanism [52,74,75,76].
Methyl salicylate	Fixed-dose hydroalcoholic and alcohol-free EO rinses: 0.060%.	Aromatic salicylate ester; formulation partitioning may affect local exposure.	Clinical evidence is mixture-level; component-level antimicrobial combinations have been tested in vitro, and isolated methyl salicylate did not reproduce the host-transcriptional effect of a complete zinc-containing formulation.	No constituent-resolved human oral exposure or molecular target engagement after rinsing was identified in this review; its independent contribution requires component-controlled studies [54,55,56].
Carvacrol	Plant-specific *Lippia sidoides* rinses: 1.0% in [68], 0.6–1.2% in the dose-ranging study [69], and 0.8% in [70]; thymol/carvacrol-dominant chemotypes.	Phenolic monoterpene; membrane partitioning and mixture effects with p-cymene and thymol.	Membrane energetics, TRPV3, PPARα/γ, and virulence-gene responses; largely constituent-level and preclinical.	Clinical *Lippia* results cannot be assigned to carvacrol alone [57,58,59,60,68,69,70,77,78].
Eugenol	Not an active ingredient in the fixed-dose four-component formulation summarized above; studied as an isolated constituent and in clove-related systems.	Phenylpropanoid with phenolic, methoxy, and allyl groups; concentration-dependent cytocompatibility.	TRPV1 activity; human dental-pulp fibroblast transcription; non-oral Nrf2-associated evidence; oral antibacterial synergy.	Human oral-cell evidence does not establish salivary biomarker modulation [79,80,81,82,83].
β-Caryophyllene	Not an active ingredient in the fixed-dose four-component formulation; caryophyllene (reported without isomer-specific attribution) constituted 3.59% of the characterized *Lippia sidoides* EO batch in one pediatric study.	Highly lipophilic bicyclic sesquiterpene; aqueous availability depends on formulation and vehicle.	Selective CB2 agonism; antagonist-sensitive effects in human gingival fibroblasts and oral mucosa epithelial cells; complementary periodontal antimicrobial/inflammatory evidence in THP-1 and bacterial systems.	Local exposure and in vivo oral CB2 engagement after rinsing were not identified in this review [70,84,85,86].
Linalool	No clinically tested formulation was identified in the direct human salivary evidence set.	Acyclic monoterpene alcohol; volatile and oxidation-sensitive.	NF-κB/MAPK-associated anti-inflammatory findings in macrophage-based and animal models.	Non-oral evidence only; oral exposure and target engagement were not identified in this review [53,87].

CB2—cannabinoid receptor 2; MAPK—mitogen-activated protein kinase; NF-κB—nuclear factor κB; Nrf2—nuclear factor erythroid 2-related factor 2; PPARα and PPARγ—peroxisome proliferator-activated receptors α and γ; TRPA1—transient receptor potential ankyrin 1; TRPM8—transient receptor potential melastatin 8; TRPV1 and TRPV3—transient receptor potential vanilloid 1 and 3.

### 4.4. Local Oral Exposure, Partitioning, and Target Engagement

The declared concentration is not necessarily the concentration attained at the biological target. During the brief rinse and subsequent clearance period, constituents may be diluted by saliva, partition among surfactant assemblies, proteins, plaque, and mucosa, and undergo volatilization or removal. Total concentration may therefore differ from the free or bioaccessible fraction [88,89,90]. Early LC–MS/MS or GC–MS sampling can characterize parent-compound washout, while plaque, GCF, or buccal-cell pellets may provide site-proximal measures. Human in situ plaque studies demonstrate formulation-level antibacterial activity [67,91], with reduced biofilm viability persisting for several hours in the study by Quintas et al. [67]; they neither quantify the exposure of each constituent nor establish mucosal target engagement. Undiluted commercial rinses reduced viability in cultured gingival and periodontal-ligament fibroblasts, with lower toxicity after dilution [92]. This does not demonstrate mucosal injury during labeled use; it shows that concentration, contact time, and vehicle must be matched when interpreting mechanistic and safety data. Target engagement requires a functional or biophysical assay in the relevant biological context. Electrophysiology or antagonist-sensitive calcium signaling may support a TRP claim; receptor antagonism or coherent target-gene responses may support CB2 or PPAR involvement; and bacterial targets require direct activity or binding assays. Docking can prioritize experiments but is not evidence of binding [93,94,95]. No human mouthrinse study identified in this review has connected quantified formulation chemistry and oral-site exposure with target-specific engagement, a salivary host-biomarker response, and a clinical outcome within one design (Figure 1).

## 5. Human Evidence: Salivary Outcomes and Clinical Context

### 5.1. Direct Human Salivary Host-Biomarker Evidence

Among the studies identified in this review, three met the review criteria for direct human salivary host-biomarker evidence [96,97,98] (Table 3). Chung [96] found no significant between-group difference in salivary IgA after a combined oral-hygiene intervention; the study separately reported a significant increase in salivary pH in the experimental group. Two studies in men with periodontal inflammation evaluated adjunctive EO rinsing after scaling and root planing (SRP): the 2015 study reported lower whole-salivary IgG at day 40 [97], and the 2016 study reported lower whole-salivary IL-1β at day 60 [98]. They assessed different single analytes, used SRP as a strong co-intervention, and did not measure formulation chemistry, oral constituent exposure, assay interference, or molecular target engagement. Salivary flow was assessed, but biomarker results were reported as concentrations rather than flow-adjusted output rates. The findings therefore do not constitute independent replication of the same molecular effect. IL-6, TNF-α, active MMP-8, MMP-9, MDA, 8-OHdG, TAC, SOD, GPx, and catalase remain candidate endpoints for future EO trials, not established EO-responsive human biomarkers.

### 5.2. Human Salivary Physiology, Biochemical Function, and Adjacent Oral-Fluid Evidence

Houghton et al. provide a human example in which oral chemosensory ligands altered salivary physiology [74]. In healthy participants, oral exposure to menthol or nonivamide increased salivary secretion; a separate repeated-exposure experiment in two men identified exploratory proteomic changes. Oral exposure to compounds pharmacologically characterized as TRP agonists increased salivary output under the tested conditions, but the study did not demonstrate receptor-specific target engagement or causal TRP dependence in vivo; the study was not a commercial EO biomarker trial and did not show that TRPM8 or TRPV1 activation reduces periodontal cytokines. Separately, Potocka et al. reported changes in salivary flow or MUC5B output after orthonasal aromatic exposure [114]; because the exposure was olfactory rather than an oral rinse, the study supports neural/secretory plausibility rather than gingival target engagement.

Product-level studies further show that salivary flow and pH are formulation- and time-dependent physiological endpoints. In 19 women with objectively documented hyposalivation, Fischman et al. used a randomized, controlled design with exaggerated exposure and measured salivary flow after an EO-containing rinse [115]. A randomized crossover pilot in 20 adults found no significant difference in whole-salivary flow or perceived dryness after one week of use of an alcohol-based versus an alcohol-free commercial rinse [116], and a larger 12-week randomized study similarly found no meaningful difference in unstimulated whole-salivary flow between an alcohol-based EO rinse and a non-alcohol comparator [117]. After an acidic challenge, Dehghan et al. found that Listerine and water both restored salivary pH to baseline immediately after rinsing, with pH higher after Listerine than water; group differences were no longer evident at 15 or 45 min [118].

Two recent randomized studies specifically evaluated EO-containing formulations with and without alcohol. A 2025 phase 4 trial in 253 adults reported increased short-term salivary flow after a single rinse compared with toothbrushing alone, while salivary pH remained in the neutral range [119]. A distinct 2026 single-day trial in 159 adults found broadly similar salivary flow by 30 min, with pH returning toward baseline across groups; alcohol content did not materially alter these endpoints under the tested conditions [120]. These studies address acute physiology and tolerability rather than inflammatory biomarker modulation, and their product-specific findings should not be extrapolated to all EO formulations. Mitsui and Harasawa compared EO, povidone-iodine, and chlorhexidine rinses in terms of salivary nitrate, nitrite, and nitrate-reducing bacteria [121]. Under the tested conditions, the EO preparation had little effect on nitrate-reducing activity. Broader literature on nitrate and the oral microbiome supports nitrate reduction as a functional ecological endpoint, but it does not justify generalizing the result of this single EO formulation to the entire class [128]. Adjacent oral-fluid evidence is similarly cautious: in a randomized crossover study, Ramberg et al. found no significant effect of an EO rinse on gingival crevicular-fluid IL-1α, IL-1β, or interleukin-1 receptor antagonist (IL-1ra) [122]. GCF is site-proximal but is not saliva, so this negative result provides contextual evidence on oral host biomarkers without qualifying as Category 1 evidence.

Category 2 studies also assessed salivary flow, pH, total protein, and alkaline phosphatase. In the longer full-mouth-disinfection trial, adjunctive EO rinsing did not alter salivary flow or pH; total salivary protein decreased in the test group, whereas salivary alkaline phosphatase did not change [102]. In a separate in vivo comparison, 2% tea tree oil and 2% lemongrass oil mouthrinses were evaluated against chlorhexidine for short-term salivary pH responses, with the largest increase reported after tea tree oil [112]. These biochemical and physiological endpoints remain distinct from targeted inflammatory or immune biomarkers.

### 5.3. Human Oral Microbial, Microbiome, and Plaque-Biofilm Evidence

Human microbial evidence spans plaque, subgingival communities, saliva, and in situ biofilms. EO formulations can change recoverable counts, viability, or community composition in human oral studies [67,68,69,70,91,123,124,125,126,129,130,131,132]; complementary in vitro microbial studies also demonstrate formulation-dependent antimicrobial effects [133,134]. A clinically meaningful ecological interpretation requires functional endpoints such as acidogenicity, nitrate reduction, protease activity, virulence expression, inflammatory potential, and recovery after treatment cessation. In supportive periodontal care, a 3-month trial found no significant change in selected subgingival taxa [123]; in another crossover study, the fixed-dose EO rinse had little effect on nitrate-reducing activity [121].

A 2026 in vitro study in which donor saliva was treated with three fluoride-containing mouthwashes, including a Listerine anti-caries product, directly measured organic-acid production and found formulation- and donor-dependent effects on acidogenic potential [135]. Because exposure occurred in vitro and the comparison concerned complete fluoride-containing products, this result supports microbial-function endpoints rather than EO-specific clinical efficacy. Short-lived reductions in recoverable bacteria and sustained changes in ecosystem function should not be treated as equivalent outcomes, and microbial findings remain distinct from human host-biomarker evidence.

Human studies report heterogeneous effects of EOs on oral microbial function and salivary microbial counts. Two randomized crossover studies found reduced plaque acidogenicity after EO rinsing following a sucrose challenge [99,100]. In full-mouth-disinfection protocols, salivary pathogen changes were mixed: a preliminary study reported a reduction in salivary *P. gingivalis* with adjunctive EO rinsing, whereas the larger follow-up study showed site- and species-dependent microbial effects [101,102]. Other salivary studies reported reductions in *S. mutans* or total microbial counts with Listerine, Melaleuca alternifolia oil, tea tree oil, or *Zingiber officinale* EO under specific conditions [104,106,110,111], but no significant EO-related reduction in cariogenic bacteria or no superiority to water was observed in other trials [105,108]. Six hours after oral hygiene, residual antimicrobial activity was detected in plaque rather than saliva, although Listerine was associated with lower salivary bacterial viability [103]. Essential-oil oral strips did not significantly alter plaque pH or *S. mutans* relative to placebo [109], and the 1% tea tree oil trial in plaque-induced gingivitis showed reductions in salivary bacterial counts with both tea tree oil and chlorhexidine, with larger reductions after chlorhexidine [113]. These findings support separating microbial counts, plaque metabolic function, and persistent ecological effects rather than treating them as equivalent evidence.

### 5.4. Formulation-Specific Clinical Evidence for Plaque and Gingivitis Control

Systematic reviews or meta-analyses synthesized controlled studies of EO-containing mouthrinses for plaque and gingival inflammation [19,20,21], and a separate systematic review compared EO and chlorhexidine rinses for the same outcomes [24]. Three six-month controlled trials evaluated daily adjunctive rinsing alongside mechanical oral hygiene and reported antiplaque and antigingivitis benefits for the commercial EO formulations tested [22,23,54].

In the Lynch trial, alcohol-containing and alcohol-free EO formulations both improved plaque, gingivitis, and bleeding relative to mechanical hygiene alone, with no statistically significant between-formulation difference within that study [54]. A later systematic review and meta-analysis directly comparing alcohol-containing with alcohol-free EO mouthwashes found a plaque-control advantage for alcohol-containing products, whereas pooled bleeding and gingival-index differences were not significant [136]. Together, these sources provide the principal clinical evidence for plaque and gingivitis control while underscoring formulation and endpoint specificity. They do not establish efficacy for the prevention or treatment of periodontitis, dental caries, oral mucosal disease, or oral cancer. A trial of a commercial rinse containing both EOs and ethyl lauroyl arginate reported a statistically lower plaque score under no-mechanical-cleaning conditions but concluded that the residual plaque made the clinical benefit questionable [137]. A 12-week trial in people with diabetes evaluated alcohol-containing and alcohol-free four-active EO formulations [127], extending clinical evidence for these formulations to a distinct population. A separate 15-day exploratory trial evaluated a 1% tea tree oil mouthwash versus chlorhexidine [113]; because it also measured unstimulated-saliva microbial counts, it is classified as Category 2 in Table 3 rather than as clinical-only evidence. Across this literature, efficacy is a formulation-level finding; constituent-specific causation and human molecular target engagement were not tested.

An isolated-constituent crossover study in 30 dental students evaluated a 0.018% menthol mouthrinse during a 5-day no-brushing period and reported reductions in plaque, gingival, and bleeding indices relative to water, although chlorhexidine produced larger reductions [107]. This finding supports formulation- and constituent-specific clinical activity but does not establish a salivary or molecular mechanism.

The clinical evidence remains concentrated in a small number of marketed formulations. Several long-term trials and recent physiology or microbiome studies report manufacturer funding, product supply, author affiliations, or other sponsor involvement [22,23,54,119,120,127,129,132]. Because this narrative review did not apply a formal risk-of-bias tool, its clinical conclusion is limited to formulation-specific efficacy and tolerability. Trials combining rinsing with SRP or full-mouth disinfection are clinically relevant. When the co-intervention is standardized across groups, between-group comparisons can estimate the incremental effect of the rinse; however, without exposure and target-specific measurements, such designs cannot establish a mouthrinse-specific molecular mechanism [101,102,138,139]. The diabetes trial [127] included no salivary host-biomarker or target-engagement endpoint.

## 6. Molecular Mechanisms: Evidence by Target and Experimental System

Mechanistic evidence is organized according to the material tested and the experimental system. Findings from isolated constituents, complete EOs, and formulated mouthrinses are not treated as interchangeable. Human oral cells or tissues and oral microbial models are presented separately from heterologous expression systems, non-oral cells, animal models, and computational studies. The latter groups can support a hypothesis but do not show that the proposed target is reached or engaged after mouthrinsing. Table 4 records both the directness of target evidence and its oral translational relevance.

### 6.1. Membranes, Biofilms, and Microbial Stress

A well-supported constituent-level mechanism is physicochemical interaction with microbial membranes. Phenolic monoterpenes and related compounds can increase permeability, dissipate proton gradients, and perturb membrane-associated functions, with activity modified by organic matter, biofilm matrix, pH, vehicle, and co-constituents [47,48,49,50,51,57,58,59,60,81]. Oral bacterial and biofilm studies support antimicrobial activity for selected oils, constituents, and formulations [45,46,67,82,91,133,134,148,149,150,151]. Component-level testing of Listerine ingredients further illustrates that activity of a mixture cannot be inferred from one constituent: combinations containing eucalyptol, methyl salicylate, and thymol were active against selected oral microorganisms in vitro, but the design did not establish an independent clinical contribution of methyl salicylate [55]. A 2026 study of carvacrol-dominant *Lippia sidoides* EO found antibacterial and antibiofilm activity against oral and bloodstream *Streptococcus sanguinis* and *Streptococcus gordonii* isolates, with cytotoxicity evaluated in human gingival fibroblasts [152]. This remains complete-EO preclinical evidence rather than proof that carvacrol alone or a commercial rinse produces the same effect. Biomass, viability, acidogenicity, matrix, virulence, and community recovery may move in different directions. Because mouthrinsing produces a short peak followed by declining exposure, microbial function at subinhibitory concentrations and post-rinse recovery are as relevant as immediate killing.

### 6.2. Microbial Virulence-Gene Expression

Oral transcriptional studies show that thymol, carvacrol, oregano or Thymus EOs, Citrus aurantium EO, and eugenol can alter virulence-associated genes in *Streptococcus mutans* or *Porphyromonas gingivalis* [143,144,145,146,147]. These are in vitro findings. Transcriptional changes must be normalized to growth and viability, confirmed at the protein or activity level, and separated from direct binding claims. They provide oral microbial hypotheses but not a human salivary mechanism.

### 6.3. TRP Channels: Strong Target Pharmacology, Incomplete Oral Engagement

TRP channels have direct constituent-level functional pharmacology. Menthol activates TRPM8; 1,8-cineole activates TRPM8 and antagonizes TRPA1; carvacrol, thymol, and eugenol activate TRPV3; and eugenol activates and desensitizes TRPV1 [64,71,72,75,76,77,79,140]. Most evidence comes from heterologous systems. The eugenol study used human odontoblast-like cells [79], and human oral exposure to menthol/nonivamide altered salivary flow and proteomic output [74], but neither result establishes anti-inflammatory target engagement by a commercial mouthrinse. Relevant human studies should compare attainable free concentrations with concentrations that produce effects in functional channel assays and report both salivary concentration and output rate.

### 6.4. Host Signaling: Oral-Cell Evidence and Non-Oral Hypotheses

β-Caryophyllene has strong receptor-level evidence: direct receptor pharmacology identifies it as a selective CB2 agonist [84], and antagonist-sensitive effects have been demonstrated in human gingival fibroblasts and human oral mucosa epithelial cells [85]. Complementary periodontal evidence from THP-1 cells and oral bacterial systems [86] supports anti-inflammatory and antimicrobial activity but does not provide equivalent CB2-antagonist validation. Even here, the human mouthrinse evidence reviewed did not identify local exposure or in vivo CB2 engagement after rinsing. A 2026 study provided formulation-level evidence in human oral cells by testing a complete zinc-containing Listerine formulation in primary human gingival fibroblasts and the HSC2 human oral squamous cell carcinoma cell line [56]. RNA sequencing, qPCR, immunoassays, phospho-STAT1 imaging, and ICP–MS showed selective attenuation of interferon-associated inflammatory transcription together with a metallothionein-rich zinc-response signature; zinc alone reproduced part of the effect, whereas isolated thymol, eucalyptol, menthol, and methyl salicylate did not reproduce the complete-formulation response [56]. The formulation was applied at 5% for 3 h, and the isolated-compound experiments were not exposure-matched to the concentrations of those actives in the 5% formulation.

The study therefore provides formulation-level oral-cell evidence but does not establish constituent-specific causation or clinically achieved target engagement. Evidence for other host pathways remains more remote. Carvacrol activated PPARα/γ reporters in non-oral cells [78]; 1,8-cineole affected defined NF-κB/JNK nodes in non-oral human cell systems [73]; thymol and linalool produced NF-κB/MAPK-associated effects in non-oral or animal models [66,87], whereas carvacrol showed anti-inflammatory activity in non-oral and animal experiments that did not establish NF-κB/MAPK pathway-node effects [65]. Eugenol altered transcripts in human dental-pulp fibroblasts [80] and has been linked to Nrf2-associated responses in non-oral systems [83]; general KEAP1–NRF2 literature provides pathway context rather than EO-specific evidence [153,154,155]. These studies justify exposure-matched oral experiments, but a change in cytokine secretion or gene expression does not by itself establish pathway dependence. Viability controls, a measured signaling node, antagonist or genetic validation, and confirmation in the relevant gingival, epithelial, immune, or salivary-gland cell type are required.

Additional oral-cell studies reinforce the dependence of host responses on cell type, concentration, and the complete EO tested. Eugenol produced cell-type- and concentration-dependent effects on IL-8 secretion in IL-1β-stimulated human gingival fibroblasts, periodontal-ligament fibroblasts, and pulp cells; inhibition was observed in pulp cells at higher concentration, whereas gingival fibroblasts did not show an anti-inflammatory response [141]. In human gingival fibroblasts, complete EOs from four Ugandan medicinal plants produced divergent effects on IL-1β-induced mediators at subcytotoxic concentrations: *Ocimum gratissimum* EO inhibited prostaglandin E2 secretion, whereas *Bidens pilosa* and *Cymbopogon nardus* EOs increased it [142]. These studies provide direct human oral-cell evidence but do not establish upstream pathway dependence, exposure after mouthrinsing, or in vivo target engagement.

### 6.5. Computational Studies

Molecular docking and related in silico analyses can prioritize candidate interactions, but they do not demonstrate binding, cellular access, or target engagement [93,144,145,156,157]. Predictions should specify ligand identity and stereochemistry, protonation state, target structure, controls, and experimental validation. In this review, computational findings remain Category 4 evidence and are not used to explain clinical mouthrinse outcomes.

## 7. Integrated Interpretation, Microbial Ecology, and Safety

### 7.1. Microbial Function and Ecological Recovery

The most defensible clinical route is indirect: a formulation changes biofilm viability, matrix, or virulence; microbial stimulation of gingival tissues is reduced; and host inflammatory or redox measures may change later. Direct host signaling remains possible for selected formulations or constituents but cannot yet be ranked against this route in humans. Community composition alone does not define benefit [158]. Trials should measure acid production, nitrate reduction, protease activity, virulence, inflammatory potential, and time to recovery after treatment. Recent in vitro donor-saliva work showing donor- and formulation-dependent differences in organic-acid production after mouthwash exposure illustrates why functional readouts can add information beyond taxonomy [135]. In this review, return toward baseline is termed recovery; “rebound” is reserved for an overshoot beyond pretreatment values.

### 7.2. Mucosal Safety, Cytocompatibility, and Vehicle

Safety depends on formulation, concentration, contact time, and user characteristics. Marketed EO rinses were generally tolerated in the evaluated trials, but burning, taste disturbance, dryness, irritation, and mucosal findings varied by product and comparator [19,20,21,22,23,24,54,113,115,116,117,118,119,120,127]. High or undiluted concentrations can be cytotoxic in oral-cell culture [92]; those experiments should not be equated with labeled use, but they justify dilution series, exposure-matched contact times, and mucosal examinations. A 2026 in vitro comparison of seven commercial mouthwashes, including Listerine Total Care, found product-dependent cytotoxicity, apoptosis, and 8-OHdG signals related to oxidative DNA damage in human gingival fibroblasts; XTT cytotoxicity was assessed across multiple concentrations and exposure periods [159]. These in vitro culture exposures, including conditions substantially longer than labeled rinsing, do not establish mucosal injury during labeled use. Ethanol acts as a solvent and may alter sensation, partitioning, and assays. In one six-month RCT, alcohol-containing and alcohol-free EO formulations were both well tolerated and showed no statistically significant difference in efficacy between the formulations [54], whereas a later meta-analysis found better plaque control with alcohol-containing EO mouthwash but no significant pooled difference for bleeding or gingival indices; taste ratings favored alcohol-free products [136]. Alcohol-containing and alcohol-free rinses should therefore be compared as complete formulations rather than treating ethanol content alone as a surrogate for efficacy or safety. Concentrated EOs should not be applied directly to oral tissues. Future trials should prespecify adverse-event definitions, oral soft-tissue examinations, discontinuations, adherence, and post-treatment ecological recovery.

## 8. Research Priorities and Study Design

### 8.1. A Minimum Human Mechanistic Trial

A next-step mechanistic trial in humans should use a randomized, double-blind, sensory-matched, vehicle-controlled design in a clinically defined population. The formulation must be chemically characterized, including methyl salicylate when present, and oral-site exposure must be measured at prespecified early time points that capture peak exposure and washout. A separate delayed sample should be collected for a prespecified host endpoint such as IL-1β or active MMP-8, with concurrent assessment of flow, plaque, bleeding, microbial function, and safety. The target-engagement assay should match the proposed mechanism and be supported by an exposure-matched oral model. This design tests the full sequence shown in Figure 1 without requiring every clinically oriented trial to become a mechanistic study.

### 8.2. Reporting and Analytical Requirements

Commercial formulation research requires explicit conflict-of-interest reporting. Sponsorship, product supply, author employment, data ownership, analytical independence, and the sponsor’s role in study design, analysis, interpretation, and publication should be stated. Because much of the long-term clinical literature is concentrated in a small number of marketed formulations, product-specific consistency should be distinguished from independent replication and class-wide generalizability. Analytical and statistical validation are integral to the biological argument. Salivary assay validation should report precision, recovery, linearity, the lower limit of quantification, freeze–thaw stability, and formulation interference; active MMP-8 assays should specify the measured molecular species, and LC–MS exposure assays should report internal standards, extraction recovery, matrix effects, and stability. Primary endpoints and the expected direction of effect should be prespecified; values below the limit of quantification and missing data should be handled according to stated rules; and omics findings should be independently validated.

### 8.3. Evidence Synthesis and Chemical Reporting Gaps

The synthesis is most transparent when direct human salivary host-biomarker evidence, adjacent human oral and salivary functional evidence, clinical efficacy, and constituent-level mechanisms remain separate. Table 5 converts the same logic into the minimum measurement domains required for a mechanistic human trial.

This framework is intended for studies seeking to attribute a human salivary response to a molecular mechanism rather than as a requirement for studies focused solely on clinical efficacy or microbial activity.

## 9. Limitations of the Evidence Base and the Review

No human study identified in this review has evaluated the full evidential chain from formulation chemistry and oral exposure to target engagement, a salivary host response, and a clinical outcome. Direct biomarker evidence consists of three heterogeneous studies, whereas much mechanistic evidence uses isolated compounds in heterologous, non-oral, animal, or computational models. A recent complete-formulation oral-cell study improves mechanistic proximity but remains in vitro and did not establish constituent-resolved exposure or in vivo target engagement. Clinical literature is concentrated in a small number of commercial formulations and includes substantial manufacturer involvement, so product-specific consistency is not equivalent to independent replication or a class effect. This was a single-author narrative review with iterative searches, no independent duplicate screening, no PRISMA flow diagram, and no formal risk-of-bias or certainty assessment. The deliberately broad mechanistic search blocks were used for structured evidence mapping and overlapped substantially with one another and with the clinical/salivary blocks; per-block retrieval counts therefore document search coverage rather than a formal flow of unique studies screened. Exact strings, eligibility rules, and selection procedures are reported to improve reproducibility, but the review should not be interpreted as a systematic estimate of effect size or certainty.

## 10. Conclusions

Formulation-specific evidence supports selected EO-containing mouthrinses as adjuncts for plaque and gingivitis control; these findings do not establish a class effect for all EOs or other plant-derived mouthrinse formulations. Direct human salivary host-biomarker evidence remains limited to three heterogeneous studies of IgA, IgG, or IL-1β and has not been independently replicated for a single analyte. Mechanistic studies provide testable hypotheses, but most do not demonstrate that constituents reach and engage the proposed target after rinsing. Progress now depends on studies that connect batch-resolved chemistry and local oral exposure with target-specific functional assays, prespecified salivary endpoints, microbial function, clinically relevant outcomes, and safety.

## Figures and Tables

**Figure 1:**
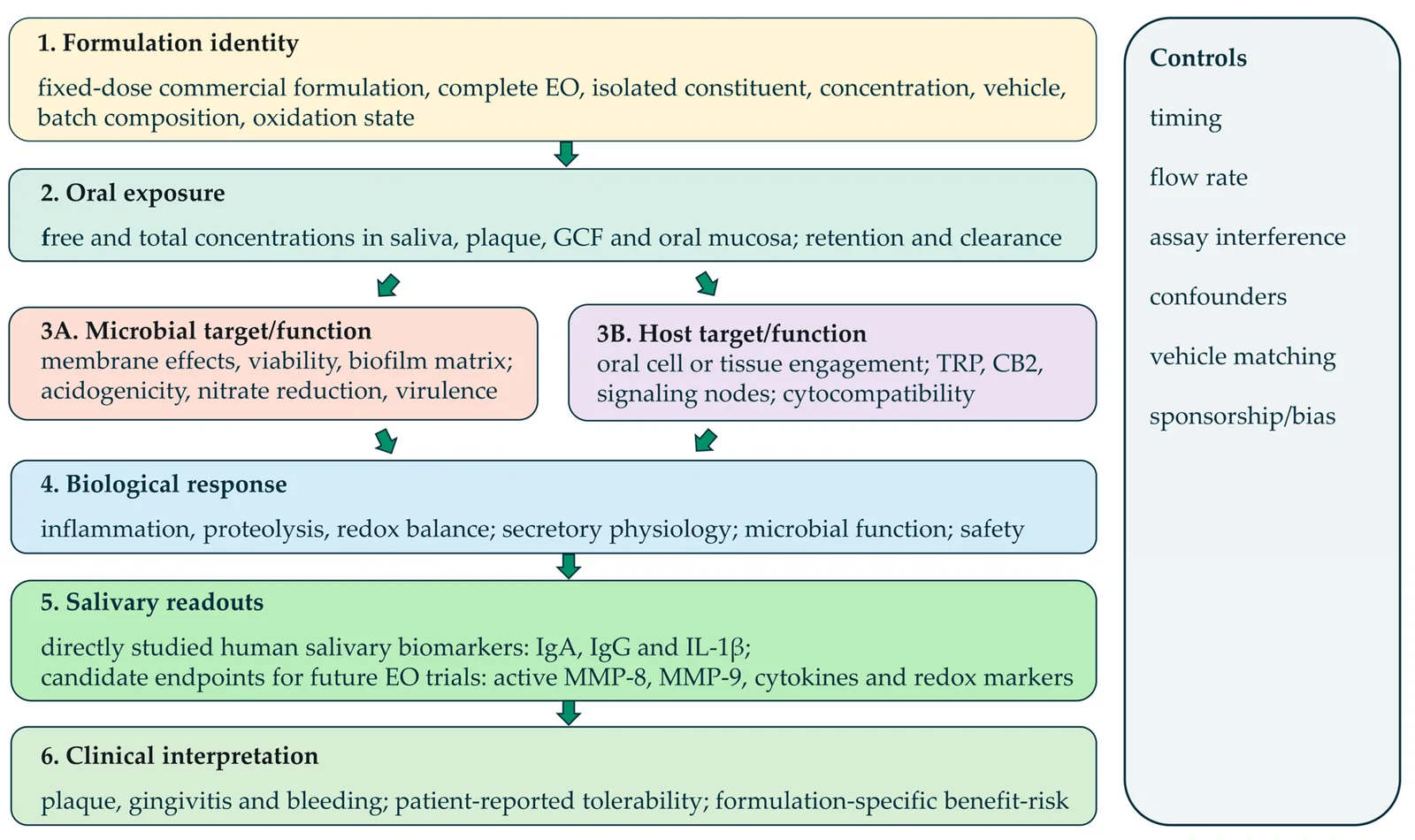
Integrated conceptual framework linking formulation identity, oral exposure, microbial and host target/functional engagement, biological responses, salivary readouts, clinical interpretation, and safety. Arrows indicate the proposed evidential sequence for mechanistic attribution and do not imply that a causal pathway has been demonstrated in humans. The right-hand column lists key interpretive controls and potential sources of bias. CB2—cannabinoid receptor 2; EO—essential oil; GCF—gingival crevicular fluid; IgA—immunoglobulin A; IgG—immunoglobulin G; IL-1β—interleukin-1β; MMP-8—matrix metalloproteinase-8; MMP-9—matrix metalloproteinase-9; TRP—transient receptor potential.

**Table 1:** Salivary endpoints for biomarker-oriented essential oil trials.

References	Principal Caveats and Limitations	Preferred Use	Biological Layer	Endpoint
[9,10,11,12,14,17]	Not EO-specific; affected by disease extent, smoking and bleeding	Whole saliva plus bleeding measures; consider GCF confirmation	Inflammatory activation; treatment response	IL-1β
[11,12,13]	Platform and molecular-species dependence	Specify whether the assay measures active enzyme, total immunoreactive protein, or both	Marker associated with collagenolytic activity	MMP-8
[9,10,11,12]	Low abundance and multiple oral sources	Secondary, hypothesis-led panel with prespecified rules for values below the limit of quantification	Broader host response and neutrophil recruitment	IL-6, TNF-α, IL-8/CXCL8
[26,27,28,29,30,31,34]	Handling and systemic confounding	Validated specific assay; record smoking, diabetes and diet	Lipid or DNA oxidative damage	MDA or 8-OHdG
[27,28,29,30,32,33,34]	Direction of change is context-dependent	Interpret with a damage marker and flow data	Antioxidant reserve and enzymatic defense	TAC, SOD/GPx/catalase
[37,45,46]	DNA-based relative abundance, biomass, and viability are not interchangeable with microbial function	Pair compositional measures with biomass/viability and functional or host endpoints	Microbial community structure and biofilm properties	Microbial composition, biomass, and viability
[35,36,37,38]	Pre-analytical handling, isolation, and normalization are major determinants	Separate discovery and targeted validation cohorts	Pathway discovery and response phenotyping	Proteome/small extracellular vesicle cargo/microbiome profiles

8-OHdG—8-hydroxy-2′-deoxyguanosine; CXCL8—C-X-C motif chemokine ligand 8; DNA—deoxyribonucleic acid; EO—essential oil; GCF—gingival crevicular fluid; GPx—glutathione peroxidase; IL—interleukin; MDA—malondialdehyde; MMP—matrix metalloproteinase; RNA—ribonucleic acid; SOD—superoxide dismutase; TAC—total antioxidant capacity; TNF-α—tumor necrosis factor α.

**Table 3:** Human studies most relevant to EO interventions and salivary or oral-exposure endpoints, with sampling and analytical details.

Study/Population (*n*)	Intervention and Sampling Time	Collection, Analytical Method, and Flow Normalization	Main Finding/Evidence Category	Confounders and Principal Limitations
Chung, 2006 [96] Community-dwelling older adults (*n* = 61)	Gingival-massage toothbrushing + EO rinse vs. gingival-massage toothbrushing. Assessments before the intervention, immediately after the 2-week intervention, and 2 weeks later.	Salivary IgA and pH; details of collection and the IgA assay were not fully available in the English-language abstract; flow normalization not reported.	No significant between-group IgA difference; salivary pH increased significantly in the experimental group as reported by the study. Category 1, with a negative between-group IgA result.	Combined hygiene intervention; older non-periodontal cohort; limited formulation and pre-analytical reporting.
Alshehri et al., 2015 [97] Systemically healthy men with periodontal inflammation (*n* = 50)	SRP + 10 mL EO rinse twice daily for 30 days vs. SRP + water; baseline and day 40.	Early-morning unstimulated whole saliva, 5 min expectoration; direct ELISA; flow recorded, but IgG was reported as a concentration rather than an output rate.	Lower follow-up whole-salivary IgG with adjunctive rinsing. Category 1.	All-male cohort; SRP co-intervention; one biomarker; allocation and analytical blinding incompletely reported; no batch chemistry or exposure assay.
Alshehri et al., 2016 [98] Systemically healthy men with periodontitis (*n* = 90)	SRP + 10 mL EO rinse twice daily for 30 days vs. SRP + water; baseline and day 60.	Early-morning unstimulated whole saliva, 5 min expectoration; IL-1β ELISA in duplicate; flow recorded, but the cytokine was reported as a concentration.	Lower follow-up whole-salivary IL-1β with adjunctive rinsing. Category 1.	Single-center all-male cohort; SRP co-intervention; one cytokine; no formulation-interference or target-engagement assay.
Houghton et al., 2020 [74] Healthy adults (WMS secretion study: 25 recruited; condition-specific *n* = 6–7; exploratory quantitative proteomics: n = 2 men)	Controlled acute oral exposure to menthol, nonivamide, cinnamaldehyde, or vehicle; the WMS secretion experiment used condition-specific participant groups (*n* = 6–7), whereas the separate proteomics experiment used repeated exposures in two participants.	Timed WMS; flow and total-protein secretion were assessed in condition-specific participant subsets; exploratory TMT quantitative MS was performed on 60 WMS samples from 2 male volunteers, with immunoassay confirmation for selected proteins.	Menthol and nonivamide increased WMS flow and total-protein secretion under the tested conditions; proteomic changes were exploratory and based on 2 participants. Category 2.	Isolated chemosensory agonists, not a commercial EO rinse or periodontal trial; interindividual and interday variability.
Zhang et al., 2004 [99] (n = 24); Wikén Albertsson et al., 2010 [100] (*n* = 20)	[99] EO rinse with/without 100 mg/kg fluoride vs. negative control, 16-day crossover; [100] EO vs. alcohol-free CHX vs. water, 16-day crossover; sucrose challenge at the end of each period.	Supragingival plaque; plaque pH measured by microelectrode/microtouch methods; metabolic acids quantified by capillary electrophoresis in [99]; salivary-flow normalization not applicable.	EO rinsing reduced plaque acidogenicity after sucrose challenge in both studies; [99] reported lower lactate, acetate, and propionate and higher plaque pH vs. control. Category 2.	Small crossover cohorts; plaque functional endpoints rather than saliva or host biomarkers; no constituent-exposure or target-engagement assay.
Cortelli et al., 2009 [101] (n = 20); Cortelli et al., 2009 [102] (*n* = 50)	One-stage full-mouth disinfection + EO rinse vs. full-mouth disinfection + placebo; follow-up to 180 days [101] and 6 months [102].	Nonstimulated saliva, tongue, and subgingival samples; PCR for periodontal pathogens; [102] additionally measured salivary flow, pH, total protein, and alkaline phosphatase.	Reference [101] reported reduced salivary *P. gingivalis* occurrence with adjunctive EO rinsing; [102] found unchanged salivary flow and pH, reduced total protein only in the EO group, and no alkaline-phosphatase change. Category 2.	Full-mouth-disinfection co-intervention; microbial effects were site- and species-dependent; general biochemical endpoints are not targeted inflammatory/immune biomarkers.
Otten et al., 2010 [103] Healthy dental/oral-hygiene students (*n* = 24)	Toothpaste alone for 2 weeks, followed by toothpaste plus one of three antimicrobial mouthrinses for 2 weeks; plaque and saliva sampled 6 h after oral hygiene.	Plaque and unstimulated saliva; bacterial concentrations and viability; experimental mixing used to assess residual antimicrobial activity.	Listerine was associated with lower salivary bacterial viability, but residual antimicrobial activity was detected in plaque rather than saliva. Category 2.	Multiple commercial mouthrinses and pooled samples; mechanistic substantivity model rather than plaque/gingivitis efficacy; no constituent-exposure measurement.
Agarwal and Nagesh, 2011 [104] (*n* = 45); Wikén Albertsson et al., 2013 [105] (*n* = 20); Nogueira et al., 2013 [106] (*n* = 26)	Short crossover/paired studies comparing Listerine or EO/tea tree oil formulations with CHX and controls.	Unstimulated saliva in [104,106] and paraffin-stimulated whole saliva in [105]; culture-based *S. mutans*/total microorganisms or mutans streptococci/lactobacilli.	Listerine reduced salivary *S. mutans* in [104]; the EO rinse did not significantly reduce cariogenic bacteria in [105]; Listerine and 2% *Melaleuca* oil produced modest short-term reductions but were less effective than CHX in [106]. Category 2.	Different ages, sampling methods, exposure schedules, and comparators; culture endpoints only; no host biomarker or constituent-exposure assay.
Ali et al., 2015 [107] Male dental students (*n* = 30)	Double-blind crossover during 5-day no-brushing periods: 0.018% menthol mouthrinse vs. 0.2% CHX vs. deionized water.	Plaque, gingival, and bleeding indices; no salivary sampling.	Menthol reduced plaque, gingival, and bleeding indices relative to water, but CHX produced larger reductions. Category 3.	Short experimental-gingivitis/no-brushing model; male dental-student cohort; isolated constituent in an ethanol-containing test solution; no molecular or salivary endpoint.
Chalhoub et al., 2016 [108] Institutionalized older adults (25 recruited; 18 completed)	Alcohol-free EO mouthwash twice daily vs. tap water; baseline, day 22, and day 45.	Plaque index, denture cleanliness, and salivary levels of several pathogens.	No significant between-group differences in clinical or microbiological outcomes. Category 2.	Feasibility pilot with substantial attrition and logistical constraints; long-term-care population; limited power.
Gemiani et al., 2018 [109] Adults (*n* = 15)	Randomized double-blind placebo-controlled crossover; oral strips containing thymol, eucalyptol, menthol, methyl salicylate, and peppermint vs. placebo.	Plaque pH and *S. mutans* colony counts before and after treatment.	No significant effect on plaque pH or *S. mutans* relative to placebo. Category 2.	Very small cohort; oral strip rather than mouthrinse; composite formulation; short exposure.
Kamath et al., 2020 [110] School children aged 8–14 years (*n* = 152)	Aloe vera, CHX, tea tree oil, or placebo mouthwash; 4 weeks of supervised rinsing plus 2-week post-treatment follow-up.	Plaque index, gingival index, and salivary *S. mutans* counts.	Tea tree oil reduced plaque, gingivitis, and salivary *S. mutans*; differences versus CHX were not significant. Category 2.	Pediatric cohort; multi-arm herbal comparison; formulation chemistry and oral constituent exposure not reported.
Bauer Faria et al., 2021 [111] Adults with fixed orthodontic appliances (*n* = 31)	Randomized crossover: 0.5% GC–MS-characterized *Zingiber* officinale EO mouthwash vs. 0.12% CHX vs. flavored-water placebo; saliva before the first rinse, 1 and 15 min after, and on day 7.	Saliva; culture-based *S. mutans* CFU/mL; clinical biofilm and bleeding assessments; flow normalization not reported.	*Zingiber* officinale EO showed antimicrobial activity against *S. mutans* and reduced biofilm/gingival bleeding, but substantivity was lower than CHX. Category 2.	Short treatment periods; orthodontic population; low flavor acceptability; no constituent-resolved oral exposure or target-engagement assay.
Manikandan et al., 2021 [112] Adults aged 26–38 years (*n* = 90)	0.2% CHX, 2% lemongrass oil, or 2% tea tree oil mouthrinse; salivary pH assessed before and after use.	Unstimulated whole saliva was collected at baseline and 1 h after rinsing; pH was measured using indicator strips.	The largest increase in salivary pH was reported after tea tree oil. Category 2.	Simple pre/post design; pH-strip measurement; no longer-term clinical or host-biomarker endpoint.
Menat et al., 2026 [113] Adults with plaque-induced gingivitis (final randomized sample, *n* = 28; 14/group)	1% (*v*/*v*) tea tree oil mouthwash vs. 0.12% CHX for 15 days; baseline and day 15.	Unstimulated saliva collected with Salivette Cortisol tubes; serial dilution and culture on Columbia blood agar and Mitis Salivarius agar; CFU/mL; flow normalization not reported.	Both groups showed reductions in plaque/gingival indices and salivary bacterial counts; clinical changes did not differ significantly between groups, whereas CHX produced greater reductions in streptococcal and total anaerobic bacterial loads. Category 2.	Small exploratory trial; short follow-up; professional scaling and oral-hygiene motivation before treatment; 1% Tween-80 in the tea tree formulation showed antimicrobial activity, limiting attribution to tea tree oil alone; no host biomarker or target-engagement measurement.
Potocka et al., 2025 [114] Healthy adults (*n* = 12)	Orthonasal aromatic exposure; acute sampling.	Salivary flow, total protein, and MUC5B output; output measures reported rather than concentration alone.	Aromatic exposure altered selected secretory outputs. Category 2.	Small cohort; olfactory rather than oral-rinse exposure; no periodontal endpoint.
Fischman et al., 2004 [115] Women with hyposalivation (*n* = 19)	Randomized controlled EO rinse vs. negative control during an exaggerated 14-day exposure protocol.	Whole-salivary flow and mucosal examination; physiological rather than host-biomarker endpoint.	Salivary flow was measured during the acute phase; oral irritation was minimal under the tested exaggerated-exposure protocol. Category 2; safety context was also assessed.	Small all-female xerostomic cohort; tolerability-focused design; product-specific inference.
Kerr et al., 2007 [116]; Kerr et al., 2015 [117] Adults (*n* = 20 pilot; *n* = 120 enrolled in the parallel trial)	Alcohol-based EO rinse vs. non-alcohol comparators; 1-week crossover pilot and 12-week parallel study.	Unstimulated whole-salivary flow and perceived dryness; no inflammatory biomarker or constituent exposure assay.	No meaningful disadvantage in salivary flow for the tested alcohol-based rinse. Category 2.	Comparator formulations differed; salivary concentration/output confounding not relevant to a host biomarker because none was measured.
Dehghan et al., 2017 [118] Healthy adults (*n* = 12)	Randomized crossover after an acidic challenge; baseline, immediate, 15 min, and 45 min measurements.	Whole-salivary pH; flow normalization not reported.	Immediately after the acidic challenge, Listerine and water restored salivary pH to baseline; pH was higher with Listerine than water, and no between-group differences were evident at 15 or 45 min. Category 2.	Small acute challenge; no constituent-resolved exposure or host biomarker.
Milleman et al., 2025 [119]; Milleman et al., 2026 [120] Adults (253 enrolled [119]; 159 completed [120])	Two distinct randomized trials of EO rinses with/without alcohol versus brushing or water controls; both included acute salivary sampling, and the 2025 trial also assessed safety through 14 days.	Salivary flow and pH; physiological endpoints, with flow measured directly.	No reduction in salivary flow or clinically concerning pH change under the tested conditions; the 2025 trial reported short-term flow increases versus brushing alone. Category 2.	Short-term endpoints; manufacturer-associated studies; no host inflammatory biomarker.
Mitsui and Harasawa, 2017 [121] Healthy non-smokers (*n* = 12)	Randomized crossover: water, fixed-dose EO rinse, povidone-iodine, or chlorhexidine; baseline and 1, 5, and 10 h after nitrate loading.	1.5 mL whole saliva; colorimetric nitrate/nitrite assay; tongue-swab *Veillonella dispar* PCR; no flow normalization.	EO rinse had little effect on nitrate-reducing activity. Category 2.	Small cohort; one formulation; nitrate-loading and dietary protocol; not an inflammatory biomarker panel.
Ramberg et al., 2016 [122] Adults (*n* = 20)	Double-blind randomized crossover EO vs. chlorhexidine vs. water; 16-day periods.	GCF IL-1α, IL-1β, and IL-1ra; GCF is site-proximal but not saliva; flow normalization not applicable.	No significant EO effect on GCF IL-1 measures. Category 2.	Small crossover study; oral-fluid rather than salivary evidence.
Cosyn et al., 2013 [123] Supportive periodontal care (*n* = 50 randomized)	EO rinse vs. placebo for 3 months.	Subgingival plaque sampling and qPCR; saliva collection and flow normalization not applicable.	No significant between-group change in selected taxa or clinical outcomes. Category 2.	Maintenance population with good baseline hygiene; selected-taxon rather than community-level functional analysis.
Balbuena et al., 1998 [124]; Pitten and Kramer, 1999 [125]; Fine et al., 2000 [126] Healthy adults: *n* = 30 [124]; *n* = 10 [125]; *n* = 29 [126]	Product-specific studies; Pitten and Kramer [125] used a crossover design with a single 20 mL rinse for 30 s and salivary bacterial counts assessed at 10, 30, and 60 min; Fine et al. [126] used 12 days of twice-daily EO rinsing.	Whole saliva; aerobic/anaerobic culture or recoverable streptococci and *Streptococcus mutans* CFU; no flow normalization.	In [125], the tested Listerine formulation was classified as having a weak, temporary salivary antibacterial effect. In [126], reductions in total streptococci and *S. mutans* were 69.9% and 75.4% in plaque and 50.8% and 39.2% in saliva. Category 2.	Older, small product-specific studies with culture endpoints; [125] measured salivary bacterial counts, not plaque or gingivitis indices.
Botelho et al., 2009 [68]; Lobo et al., 2011 [69]; Lobo et al., 2014 [70] *Lippia sidoides* studies: *n* = 55 [68]; *n* = 37 in the dose-ranging study plus *n* = 11 in the confirmatory comparison [69]; *n* = 81 [70]	*Lippia* rinse protocols differed across studies: 1% twice daily for 1 week in [68]; single-dose 0.6–1.2% dose-ranging mouthrinses with an additional 0.8% confirmatory comparison in [69]; and 0.8% once daily for 5 consecutive days in [70], with follow-up through day 365 in [70].	Whole saliva; culture-based mutans streptococci/*S. mutans* counts (CFU/mL); no salivary-flow normalization reported.	Formulation- and time-dependent microbial reductions; durable effects were inconsistent for mouthwash formulations. Category 2.	Plant-specific chemotypes; pediatric and adult populations and active comparators; mechanical hygiene differed across studies.
Quintas et al., 2017 [67] Healthy adults (*n* = 18 analyzed)	Randomized crossover of traditional EO, alcohol-free EO, and water; 30 s and 1, 3, 5, and 7 h after one rinse, plus a 4-day model.	In situ plaque-like biofilm on disks; LIVE/DEAD staining and confocal microscopy; saliva collection/flow normalization not applicable.	Reduced biofilm viability persisted for up to 7 h after both EO formulations (described by the study as substantivity). Category 2.	No constituent concentration measured at the biofilm; no host biomarker; dental-student cohort.
Milleman et al., 2026 [127] People with diabetes (*n* = 154)	Examiner-blind 12-week RCT of alcohol-containing and alcohol-free EO rinses vs. negative control.	Plaque, gingivitis, bleeding, and safety; no salivary sampling or normalization.	Improved clinical outcomes for the tested formulations. Category 3.	Manufacturer-associated; clinical-only evidence; no host biomarker or molecular target engagement.

CFU—colony-forming unit; CHX—chlorhexidine; EO—essential oil; GCF—gingival crevicular fluid; IgA—immunoglobulin A; IgG—immunoglobulin G; IL—interleukin; MUC5B—mucin 5B; PCR—polymerase chain reaction; qPCR—quantitative polymerase chain reaction; SRP—scaling and root planing; TMT—tandem mass tag; WMS—whole-mouth saliva.

**Table 4:** Mechanistic evidence characterization for proposed targets and pathways relevant to essential oil sources and constituents.

Evidence Entity (Isolated Constituent, Whole EO, or Formulation)	Target/Pathway	Key Experimental System	Target/Pathway Evidence	Human Oral Engagement	References
Menthol; 1,8-cineole	Menthol—TRPM8; 1,8-cineole—TRPM8/TRPA1	1,8-Cineole: heterologous human TRPM8/TRPA1 channel systems and non-oral animal inflammatory models; menthol: heterologous TRPM8 systems plus salivary responses in healthy humans after oral menthol exposure	Direct channel pharmacology for each constituent in heterologous systems; [74] provides human oral sensory/secretory relevance for menthol exposure only, not receptor-specific engagement in vivo	No periodontal/oral target engagement after EO mouthrinse use was identified in the cited evidence	[71,72,74,75,76]
Carvacrol; thymol; eugenol	TRPV3; weak TRPM8 activation by thymol in a heterologous assay	Heterologous channel expression/electrophysiology; carvacrol mutagenesis in [77]; additional native epithelial-cell responses in [140]	Direct functional TRPV3 evidence; carvacrol activation determinants were tested by mutagenesis; thymol showed only limited TRPM8 activity	Not demonstrated in the cited studies; oral tissue exposure and downstream effects remain unresolved	[64,77,140]
Eugenol	TRPV1	Human odontoblast-like cells	Functional TRPV1 activation and exposure-dependent desensitization in human oral cells	Salivary inflammatory relevance was not demonstrated in the cited study	[79]
β-Caryophyllene	CB2	Direct receptor pharmacology; human gingival fibroblasts and human oral mucosa epithelial cells; complementary THP-1/oral bacterial experiments	Direct CB2 pharmacology [84]; AM630-sensitive effects in human oral cells [85]; complementary periodontal activity without equivalent CB2-antagonist validation [86]	Exposure after mouthrinsing in humans was not quantified, nor was in vivo CB2 engagement after rinsing identified in the cited evidence	[84,85,86]
Carvacrol	PPARα/γ	Non-oral reporter and gene-expression systems	PPAR reporter and gene-expression pathway evidence in non-oral cells	Intracellular exposure and PPAR engagement in human oral tissues were not established in the cited evidence	[78]
1,8-Cineole	NF-κB/JNK	Human cell systems	Measured NF-κB/JNK pathway-node effects in human cell systems	Not exposure-matched to mouthrinse use	[73]
Eugenol	Inflammation- and apoptosis-related genes; Nrf2	Human dental-pulp fibroblasts; non-oral cultured cells	Human oral-cell transcriptional evidence; Nrf2-associated evidence is non-oral	Engagement after mouthrinse use was not demonstrated in the cited evidence	[80,83]
Eugenol; complete EOs from *Ocimum gratissimum*, *Bidens pilosa*, *Cymbopogon nardus*, and *Zanthoxylum chalybeum*	IL-1β-induced inflammatory mediator secretion	Human gingival fibroblasts, periodontal-ligament fibroblasts, and pulp cells; cytokine/prostaglandin immunoassays with cytotoxicity controls	Eugenol showed cell-type- and concentration-dependent IL-8 effects [141]; plant EOs produced divergent IL-6/IL-8/prostaglandin E2 responses, including inhibition of prostaglandin E2 by *O. gratissimum* EO [142]	Direct human oral-cell evidence; no mouthrinse exposure measurement or in vivo pathway/target engagement	[141,142]
Isolated carvacrol, eugenol and thymol; oregano EOs (*Origanum vulgare* and *O. heracleoticum*); *Thymus* spp. EOs; *Citrus aurantium* EO	Microbial virulence, biofilm, and stress-response networks	*Streptococcus mutans*/*Porphyromonas gingivalis*; in vitro; qRT-PCR and phenotypic assays; selected docking analyses	In vitro functional/transcriptional oral microbial evidence; selected docking analyses used for prioritization	No human salivary mechanism demonstrated; transcription does not establish direct target engagement	[143,144,145,146,147]

CB2—cannabinoid receptor 2; CXCL10 and CXCL11—C-X-C motif chemokine ligands 10 and 11; EO—essential oil; ICP–MS—inductively coupled plasma mass spectrometry; JNK–c—Jun N-terminal kinase; NF-κB—nuclear factor κB; Nrf2—nuclear factor erythroid 2-related factor 2; PPARα and PPARγ—peroxisome proliferator-activated receptors α and γ; qPCR—quantitative polymerase chain reaction; qRT-PCR—quantitative reverse-transcription polymerase chain reaction; RNA-seq—RNA sequencing; STAT1—signal transducer and activator of transcription 1; TRP—transient receptor potential; TRPA1—transient receptor potential ankyrin 1; TRPM8—transient receptor potential melastatin 8; TRPV1 and TRPV3—transient receptor potential vanilloid 1 and 3.

**Table 5:** Minimum package for a target-engagement trial of an EO-containing mouthrinse.

Domain	Minimum Measurement	Rationale
Formulation identity	Validated quantitative GC–MS/LC–MS; vehicle, pH, ethanol/surfactants, stability, and batch consistency	Prevents attribution to an undefined mixture
Local exposure	Serial saliva and GCF; parent compounds and metabolites; free or bioaccessible fraction; clearance; plaque or cell partitioning	Shows whether a biologically relevant concentration is attained
Target engagement	Target-specific functional or biophysical assay, antagonist or genetic validation, exposure-matched ex vivo model	Separates plausible pharmacology from mechanism in the oral context
Microbial response	Viability/biomass plus community-level and functional RNA-, metabolite-, and enzyme-based endpoints, with washout and post-discontinuation recovery sampling	Distinguishes bactericidal effects from ecological or virulence modulation and differentiates recovery from a true rebound
Host response	IL-1β, assay-verified active MMP-8, defined redox pair; pathway marker in oral cells; viability and barrier measures	Links engagement to a salivary pharmacodynamic effect
Clinical response	Plaque, bleeding, gingivitis/periodontal indices, tolerability and adherence	Establishes clinical interpretability
Analysis	Baseline-adjusted between-group effects, confidence intervals, multiplicity, mediation, prespecified subgroups	Reduces false-positive mechanistic narratives

EO—essential oil; GCF—gingival crevicular fluid; GC–MS—gas chromatography–mass spectrometry; IL-1β—interleukin-1β; LC–MS—liquid chromatography–mass spectrometry; MMP-8—matrix metalloproteinase-8; RNA—ribonucleic acid.

## Data Availability

No new data were created or analyzed in this study. Data sharing is not applicable to this article.

## Supplementary Materials

The following supporting information can be downloaded at https://www.mdpi.com/article/10.3390/molecules31193417/s1. Table S1: Reproducible literature-search strategy, eligibility criteria, and study-selection procedures.

## Abbreviations

8-OHdG	8-hydroxy-2′-deoxyguanosine;
ABTS	2,2′-azino-bis(3-ethylbenzothiazoline-6-sulfonic acid);
ARE	antioxidant response element;
CB2	cannabinoid receptor 2;
CFU	colony-forming unit;
CHX	chlorhexidine;
COX-2	cyclooxygenase-2;
CRediT	Contributor Roles Taxonomy;
CXCL8	C-X-C motif chemokine ligand 8;
DOI	digital object identifier;
DPPH	2,2-diphenyl-1-picrylhydrazyl;
ELISA	enzyme-linked immunosorbent assay;
EO	essential oil;
ERK	extracellular signal-regulated kinase;
GCF	gingival crevicular fluid;
GC–MS	gas chromatography–mass spectrometry;
GPx	glutathione peroxidase;
HSC2	human oral squamous cell carcinoma cell line;
IgA	immunoglobulin A;
IgG	immunoglobulin G;
IκB	inhibitor of κB;
IL	interleukin;
IL-1ra	interleukin-1 receptor antagonist;
JNK	c-Jun N-terminal kinase;
Keap1	Kelch-like ECH-associated protein 1;
LC–MS	liquid chromatography–mass spectrometry;
LC–MS/MS	liquid chromatography–tandem mass spectrometry;
MAPK	mitogen-activated protein kinase;
MDA	malondialdehyde;
MMP	matrix metalloproteinase;
MUC5B	mucin 5B;
NF-κB	nuclear factor κB;
NQO1	NAD(P)H quinone dehydrogenase 1;
Nrf2	nuclear factor erythroid 2-related factor 2;
PMID	PubMed identifier;
PPAR	peroxisome proliferator-activated receptor;
PRISMA	Preferred Reporting Items for Systematic Reviews and Meta-Analyses;
pSTAT1	phosphorylated signal transducer and activator of transcription 1;
qPCR	quantitative polymerase chain reaction;
qRT-PCR	quantitative reverse-transcription polymerase chain reaction;
RCT	randomized controlled trial;
SANRA	Scale for the Assessment of Narrative Review Articles;
SOD	superoxide dismutase;
SRP	scaling and root planing;
TAC	total antioxidant capacity;
THP-1	human monocytic leukemia cell line;
TNF-α	tumor necrosis factor α;
TRP	transient receptor potential;
TRPA1	transient receptor potential ankyrin 1;
TRPM8	transient receptor potential melastatin 8;
TRPV1	transient receptor potential vanilloid 1;
TRPV3	transient receptor potential vanilloid 3;
XTT	(sodium 3′-[1-[(phenylamino)-carbony]-3,4-tetrazolium]-bis(4-methoxy-6-nitro)benzene-sulfonic acid hydrate).
