# Supplementary material for: Essential Oil-Containing Mouthrinses for Plaque and Gingivitis Control: Formulation Chemistry, Human Salivary Biomarkers, Mechanistic Evidence, and Research Priorities
Source: Molecules. 2026 Sep 25;31(19):3417. doi: 10.3390/molecules31193417 (PMC13666469; doi:10.3390/molecules31193417)
Supplement: Supplementary file 1 [file molecules-31-03417-s001.zip › Supplementary Material/Supplementary Material_Table S1.pdf]

Table S1. Reproducible literature-search strategy, eligibility criteria, and study-selection procedures.

Item	Operational Specification / Exact Search String
Review scope and cutoff	The narrative synthesis focused on the clinical use of EO-containing mouthrinses for plaque and gingivitis control. Formulation chemistry, direct human salivary host biomarkers, adjacent oral/salivary physiology and microbial function, periodontal context, safety, and mechanistic evidence were included to explain or delimit the clinical evidence. The review does not claim broad efficacy across oral diseases. Final search update: 9 September 2026; eligible records were published or available online by that date.
Primary bibliographic sources	PubMed/MEDLINE and Web of Science Core Collection (WoS CC). Publisher sites, DOI/Crossref metadata, and reference lists were used for full-text retrieval, bibliographic verification, and backward citation tracking; they were not treated as separate bibliographic databases.
Search architecture and role of blocks	Six complementary blocks were run separately in each primary database: (1) clinical plaque/gingivitis; (2) saliva/biomarkers/physiology; (3) oral mechanistic and microbial-function evidence; (4) direct molecular targets (TRP/CB2/PPAR); (5) host signaling (NF-κB/MAPK/Nrf2); and (6) a dedicated methyl-salicylate supplementary targeted search. The clinical and salivary blocks identified the core human evidence. The broader oral/microbial, direct-target, and host-signaling blocks were used for structured mechanistic evidence mapping and contextualization. The methyl-salicylate block was used to test for constituent-specific oral evidence. Retrieval counts below are block-level counts; blocks overlap and must not be summed as unique studies.
PubMed 1: Clinical plaque/gingivitis<br>Results: 304	((Listerine[tiab] OR "Lippia sidoides"[tiab] OR "essential oil mouthrinse*"[tiab] OR "essential oil mouthwash*"[tiab] OR "essential oil-containing mouthrinse*"[tiab] OR "essential oil-containing mouthwash*"[tiab] OR ("essential oils"[tiab] AND (mouthrinse*[tiab] OR mouthwash*[tiab]))) AND (plaque[tiab] OR gingiv*[tiab] OR antiplaque[tiab] OR antigingivitis[tiab] OR "gingival bleeding"[tiab])) OR (("essential oil"[tiab] OR "essential oils"[tiab]) AND (mouthwash*[tiab] OR mouthrinse*[tiab] OR

	"mouth rinse*"[tiab] OR "oral rinse*"[tiab]) AND ("systematic review"[tiab] OR meta-analy*[tiab] OR meta-regression[tiab]))
PubMed 2: Saliva/biomarkers/physiology Results: 168	((("Oils, Volatile"[Mesh] AND "Mouthwashes"[Mesh]) OR (("essential oil"[tiab] OR "essential oils"[tiab] OR Listerine[tiab] OR "Lippia sidoides"[tiab]) AND (mouthrinse*[tiab] OR mouthwash*[tiab] OR "mouth rinse"[tiab] OR "mouth rinses"[tiab] OR "oral rinse"[tiab] OR "oral rinses"[tiab])))) AND ("Saliva"[Mesh] OR saliva*[tiab] OR "Gingival Crevicular Fluid"[Mesh] OR "gingival crevicular fluid"[tiab] OR GCF[tiab] OR xerostom*[tiab] OR hyposalivat*[tiab] OR "dry mouth"[tiab])
PubMed 3: Oral mechanistic and microbial function Results: 664 exported rows; 655 unique PMIDs	(("Oils, Volatile"[Mesh] OR "essential oil"[tiab] OR "essential oils"[tiab] OR Listerine[tiab] OR eucalyptol[tiab] OR "1,8-cineole"[tiab] OR thymol[tiab] OR "methyl salicylate"[tiab] OR menthol[tiab] OR carvacrol[tiab] OR eugenol[tiab] OR "beta-caryophyllene"[tiab] OR caryophyllene[tiab] OR linalool[tiab] OR "Lippia sidoides"[tiab] OR "Origanum vulgare"[tiab] OR "Origanum heracleoticum"[tiab] OR "Thymus vulgaris"[tiab]) AND ("Streptococcus mutans"[tiab] OR "Porphyromonas gingivalis"[tiab] OR "Fusobacterium nucleatum"[tiab] OR "oral biofilm"[tiab] OR "oral biofilms"[tiab] OR "dental plaque"[tiab] OR "oral bacteria"[tiab] OR "plaque bacteria"[tiab] OR "plaque microorganism"[tiab] OR "plaque microorganisms"[tiab] OR "oral pathogen"[tiab] OR "oral pathogens"[tiab] OR periodontopathogen*[tiab] OR "gingival fibroblast"[tiab] OR "gingival fibroblasts"[tiab] OR "periodontal ligament"[tiab] OR "oral epithelial"[tiab] OR "oral keratinocyte"[tiab] OR "oral keratinocytes"[tiab] OR odontoblast*[tiab] OR "dental pulp"[tiab]) AND (biofilm*[tiab] OR virulence[tiab] OR adhes*[tiab] OR acidogenic*[tiab] OR protease*[tiab] OR "membrane permeability"[tiab] OR "membrane potential"[tiab] OR cytotox*[tiab] OR viability[tiab] OR antibacter*[tiab] OR antimicrobial*[tiab] OR "gene expression"[tiab] OR coaggregat*[tiab] OR endotoxin[tiab])) OR (("Oils, Volatile"[Mesh] OR "essential oil"[tiab] OR "essential oils"[tiab] OR Listerine[tiab] OR "Lippia sidoides"[tiab]) AND (mouthrinse*[tiab] OR mouthwash*[tiab] OR "oral rinse"[tiab] OR "oral rinses"[tiab] OR saliva*[tiab] OR plaque[tiab] OR "oral biofilm"[tiab] OR "oral biofilms"[tiab]) AND (microbiom*[tiab] OR microbiota[tiab] OR "16S"[tiab] OR metagenom*[tiab] OR

	acidogenic*[tiab] OR "acid production"[tiab] OR "nitrate reduction"[tiab] OR "nitrate-reducing"[tiab] OR protease*[tiab] OR virulence[tiab]))
PubMed 4: Direct molecular targets (TRP/CB2/PPAR) Results: 696	("menthol TRPM8"[tiab:~10] OR "1,8-cineole TRPM8"[tiab:~10] OR "1,8-cineol TRPM8"[tiab:~10] OR "eucalyptol TRPM8"[tiab:~10] OR "1,8-cineole TRPA1"[tiab:~10] OR "1,8-cineol TRPA1"[tiab:~10] OR "eucalyptol TRPA1"[tiab:~10] OR "carvacrol TRPV3"[tiab:~10] OR "thymol TRPV3"[tiab:~10] OR "thymol TRPM8"[tiab:~10] OR "eugenol TRPV1"[tiab:~10] OR "eugenol TRPV3"[tiab:~10] OR "beta-caryophyllene CB2"[tiab:~10] OR "caryophyllene CB2"[tiab:~10] OR "beta-caryophyllene CNR2"[tiab:~10] OR "caryophyllene CNR2"[tiab:~10] OR "carvacrol PPAR"[tiab:~10]) OR (carvacrol[tiab] AND (PPARalpha[tiab] OR PPARgamma[tiab] OR "PPAR alpha"[tiab] OR "PPAR gamma"[tiab])) OR (("beta-caryophyllene"[tiab] OR caryophyllene[tiab]) AND (CB2[tiab] OR CNR2[tiab] OR "cannabinoid receptor 2"[tiab])) OR ((carvacrol[tiab] OR thymol[tiab] OR eugenol[tiab]) AND TRPV3[tiab])
PubMed 5: Host signaling (NF-κB/MAPK/Nrf2) Results: 683	(eucalyptol[tiab] OR "1,8-cineole"[tiab] OR "1,8-cineol"[tiab] OR thymol[tiab] OR "methyl salicylate"[tiab] OR menthol[tiab] OR carvacrol[tiab] OR eugenol[tiab] OR "beta-caryophyllene"[tiab] OR caryophyllene[tiab] OR linalool[tiab]) AND ("NF-kappa B"[tiab] OR "NF-kappaB"[tiab] OR NFKB[tiab] OR NFkB[tiab] OR p65[tiab] OR JNK[tiab] OR "c-Jun N-terminal kinase"[tiab] OR "c-Jun N-terminal kinases"[tiab] OR MAPK[tiab] OR "mitogen-activated protein kinase"[tiab] OR "mitogen-activated protein kinases"[tiab] OR p38[tiab] OR "p38 MAPK"[tiab] OR ERK[tiab] OR "extracellular signal-regulated kinase"[tiab] OR "extracellular signal-regulated kinases"[tiab] OR "I-kappa B"[tiab] OR IkappaB[tiab] OR "I-kappaB"[tiab] OR Nrf2[tiab] OR NRF2[tiab] OR NFE2L2[tiab] OR Keap1[tiab] OR "antioxidant response element"[tiab])
PubMed 6: Methyl salicylate validation/rescue Results: 81	("methyl salicylate"[nm] OR "methyl salicylate"[tiab] OR methylsalicylate[tiab]) AND (mouthwash*[tiab] OR mouthrinse*[tiab] OR "mouth rinse"[tiab] OR "mouth rinses"[tiab] OR "oral rinse"[tiab] OR "oral rinses"[tiab] OR oral[tiab] OR dental[tiab] OR saliva*[tiab] OR plaque[tiab] OR gingiv*[tiab] OR periodont*[tiab] OR biofilm*[tiab] OR fibroblast*[tiab] OR "oral bacteria"[tiab] OR "oral pathogen*"[tiab])

Web of Science Core Collection search field	All six Web of Science Core Collection (WoS CC) searches were run in the Topic field (TS). The fielded search strings below are reported in the complete Advanced Search syntax, i.e., TS=(...), as archived at the time of the search. To reproduce a search in the standard WoS search form, select Topic from the field menu and paste only the expression contained within TS=(...); the interface adds the TS= field tag automatically. Do not paste the TS= prefix into a search box already set to Topic, because this may generate a nested expression such as TS=(TS=(...)) and return a syntax error. Alternatively, the complete TS=(...) string may be pasted directly into the Advanced Search/Query Builder. Retrieval counts are the raw block-level results obtained on 9 September 2026 and may change with subsequent database updates.
WoS CC 1: Clinical plaque/gingivitis<br>Results: 454	TS=(((Listerine OR "Lippia sidoides" OR "essential oil mouthrinse" OR "essential oil mouthrinses" OR "essential oil mouthwash" OR "essential oil mouthwashes" OR "essential oil-containing mouthrinse" OR "essential oil-containing mouthrinses" OR "essential oil-containing mouthwash" OR "essential oil-containing mouthwashes" OR ("essential oils" AND (mouthrinse* OR mouthwash*))) AND (plaque OR gingiv* OR antiplaque OR antigingivitis OR "gingival bleeding")) OR (("essential oil" OR "essential oils") AND (mouthwash* OR mouthrinse* OR "mouth rinse" OR "mouth rinses" OR "oral rinse" OR "oral rinses") AND ("systematic review" OR meta-analy* OR meta-regression)))
WoS CC 2: Saliva/biomarkers/physiology<br>Results: 181	TS=((("essential oil" OR "essential oils" OR Listerine OR "Lippia sidoides") AND (mouthrinse* OR mouthwash* OR "mouth rinse" OR "mouth rinses" OR "oral rinse" OR "oral rinses")) AND (saliva* OR "gingival crevicular fluid" OR GCF OR xerostom* OR hyposalivat* OR "dry mouth"))
WoS CC 3: Oral mechanistic and microbial function<br>Results: 1197	TS=((("essential oil" OR "essential oils" OR Listerine OR eucalyptol OR "1,8-cineole" OR "1,8-cineol" OR thymol OR "methyl salicylate" OR menthol OR carvacrol OR eugenol OR "beta-caryophyllene" OR caryophyllene OR linalool OR "Lippia sidoides" OR "Origanum vulgare" OR "Origanum heracleoticum" OR "Thymus vulgaris") AND ("Streptococcus mutans" OR "Porphyromonas gingivalis" OR "Fusobacterium nucleatum" OR "oral biofilm" OR "oral biofilms" OR "dental plaque" OR "oral bacteria" OR "plaque bacteria" OR "plaque microorganism" OR "plaque microorganisms" OR "oral pathogen" OR "oral pathogens" OR

	periodontopathogen* OR "gingival fibroblast" OR "gingival fibroblasts" OR "periodontal ligament" OR "oral epithelial" OR "oral keratinocyte" OR "oral keratinocytes" OR odontoblast* OR "dental pulp") AND (biofilm* OR virulence OR adhes* OR acidogenic* OR protease* OR "membrane permeability" OR "membrane potential" OR cytotox* OR viability OR antibacter* OR antimicrobial* OR "gene expression" OR coaggregat* OR endotoxin)) OR (("essential oil" OR "essential oils" OR Listerine OR "Lippia sidoides") AND (mouthrinse* OR mouthwash* OR "oral rinse" OR "oral rinses" OR saliva* OR plaque OR "oral biofilm" OR "oral biofilms") AND (microbiom* OR microbiota OR 16S OR metagenom* OR acidogenic* OR "acid production" OR "nitrate reduction" OR "nitrate-reducing" OR protease* OR virulence)))
WoS CC 4: Direct molecular targets (TRP/CB2/PPAR) Results: 876	TS=(((menthol NEAR/10 TRPM8) OR ("1,8-cineole" NEAR/10 TRPM8) OR ("1,8-cineol" NEAR/10 TRPM8) OR (eucalyptol NEAR/10 TRPM8) OR ("1,8-cineole" NEAR/10 TRPA1) OR ("1,8-cineol" NEAR/10 TRPA1) OR (eucalyptol NEAR/10 TRPA1) OR (carvacrol NEAR/10 TRPV3) OR (thymol NEAR/10 TRPV3) OR (thymol NEAR/10 TRPM8) OR (eugenol NEAR/10 TRPV1) OR (eugenol NEAR/10 TRPV3) OR ("beta-caryophyllene" NEAR/10 CB2) OR (caryophyllene NEAR/10 CB2) OR ("beta-caryophyllene" NEAR/10 CNR2) OR (caryophyllene NEAR/10 CNR2) OR (carvacrol NEAR/10 PPAR*)) OR (carvacrol AND (PPARalpha OR PPARgamma OR "PPAR alpha" OR "PPAR gamma")) OR (("beta-caryophyllene" OR caryophyllene) AND (CB2 OR CNR2 OR "cannabinoid receptor 2")) OR ((carvacrol OR thymol OR eugenol) AND TRPV3))
WoS CC 5: Host signaling (NF-κB/MAPK/Nrf2) Results: 984	TS=((eucalyptol OR "1,8-cineole" OR "1,8-cineol" OR thymol OR "methyl salicylate" OR menthol OR carvacrol OR eugenol OR "beta-caryophyllene" OR caryophyllene OR linalool) AND ("NF-kappa B" OR "NF-kappaB" OR NFKB OR NFkB OR p65 OR JNK OR "c-Jun N-terminal kinase" OR "c-Jun N-terminal kinases" OR MAPK OR "mitogen-activated protein kinase" OR "mitogen-activated protein kinases" OR p38 OR "p38 MAPK" OR ERK OR "extracellular signal-regulated kinase" OR "extracellular signal-regulated kinases" OR "I-kappa B" OR IkappaB OR "I-kappaB" OR Nrf2 OR NRF2 OR NFE2L2 OR Keap1 OR "antioxidant response element"))

WoS CC 6: Methyl salicylate validation<br>Results: 106	TS=(("methyl salicylate" OR methylsalicylate) AND (mouthwash* OR mouthrinse* OR "mouth rinse" OR "mouth rinses" OR "oral rinse" OR "oral rinses" OR oral OR dental OR saliva* OR plaque OR gingiv* OR periodont* OR biofilm* OR fibroblast* OR "oral bacteria" OR "oral pathogen" OR "oral pathogens"))
Search filters	No language filter and no "Humans" filter were applied in the database interfaces. Animal-only and non-oral evidence was not removed by an automated search filter because selected mechanistic blocks intentionally mapped non-human or non-oral models relevant to stated oral hypotheses. Eligibility and evidence level were determined during study selection.
Human study inclusion	Human studies were eligible when they evaluated an EO-containing oral formulation or a named EO constituent and reported any of the following: a salivary host marker; salivary physiology or biochemistry; an oral microbial or biofilm endpoint; a GCF marker; a plaque, gingival, or periodontal outcome; or a safety outcome.
Mechanistic study inclusion	Mechanistic primary studies were eligible when they tested a named EO, formulation, or constituent in an oral microorganism, a human oral cell or tissue model, another defined biological model, a purified target, or a computational system relevant to a stated oral hypothesis. Systematic reviews and methodological papers were retained for clinical context, assay interpretation, and narrative-review methods.
Exclusion	Non-EO herbal mixtures without chemical definition; non-oral uses lacking a direct mechanistic link to the oral question; duplicate reports; conference abstracts without sufficient methods; clinical indices misclassified as direct salivary evidence; GCF misclassified as saliva; and docking-only claims presented as evidence of binding or target engagement.
Study selection and duplicate handling	The sole author screened titles and abstracts, assessed potentially eligible full texts, and performed backward citation tracking from relevant reviews and primary studies. Selection was iterative because the review was designed to connect clinical, salivary, chemical, microbial, and mechanistic domains rather than to estimate a pooled effect. Overlapping citations across databases and blocks were consolidated using PMID, DOI, normalized title, and manual bibliographic verification where necessary. The PubMed oral/microbial export contained 664 rows but 655 unique PMIDs (9 duplicate export rows).

	No independent duplicate screening or PRISMA flow diagram was used; block-level retrieval counts are therefore reported for reproducibility rather than as a PRISMA study-flow denominator.
Evidence categories	Category 1: direct human salivary inflammatory or immune host-biomarker evidence, defined as human intervention studies in which a targeted salivary inflammatory or immune analyte was evaluated as a treatment-response endpoint. Category 2: adjacent human oral or salivary functional and biochemical evidence, including salivary secretion, pH, general biochemical measures, exploratory proteomic responses, oral microbial or biofilm endpoints, and GCF markers. Category 3: clinical outcomes without a Category 1 or 2 endpoint. Category 4: mechanistic or exploratory evidence. Within Category 4, isolated constituents, complete EOs, and commercial formulations were kept separate, and human oral cells or tissues were distinguished from heterologous, non-oral, animal, microbial, purified target, and computational models.
Mechanistic inference rules	Direct target evidence required a defined protein or channel together with a functional or biophysical assay. Pathway evidence required a measured signaling step; a change in a secreted cytokine alone was not treated as proof of the upstream mechanism. For microbial studies, growth inhibition was distinguished from altered virulence-gene expression and from direct binding to a predicted protein target. Docking was treated as hypothesis-generating rather than evidence of binding or target engagement.
Appraisal and synthesis limits	SANRA was used only as an appraisal checklist for the quality of the completed narrative review; it did not define eligibility and is not presented as a reporting guideline. No formal risk-of-bias tool, certainty grading, or quantitative meta-analysis was applied. Study sponsorship, model proximity to the oral question, formulation specificity, and the permitted level of inference were considered narratively and reported as limitations.

Note. An asterisk (*) denotes truncation. Search blocks were run separately and overlap substantially; raw block-level counts should not be summed as unique studies. GCF–gingival crevicular fluid; EO–essential oil; WoS CC–Web of Science Core Collection; TRP–transient receptor potential; CB2–cannabinoid receptor 2; PPAR–peroxisome proliferator-activated receptor; NF-κB–nuclear factor κB; MAPK–mitogen-activated protein kinase; Nrf2–nuclear factor erythroid 2-related factor 2.
